# Association of Body Mass Index with Outcomes in Patients with Heart Failure with Reduced Ejection Fraction (HFrEF)

**DOI:** 10.3390/nu16152473

**Published:** 2024-07-30

**Authors:** Michał Czapla, Stanisław Surma, Adrian Kwaśny, Łukasz Lewandowski

**Affiliations:** 1 Department of Emergency Medical Service, Wrocław Medical University, 51-616 Wrocław, Poland; 2 Group of Research in Care (GRUPAC), Faculty of Health Sciences, University of La Rioja, 26006 Logroño, Spain; 3 Institute of Heart Diseases, University Hospital, 50-566 Wrocław, Poland; 4 Department of Internal Medicine and Clinical Pharmacology, Medical University of Silesia, 40-752 Katowice, Poland; stanislaw.surma@ptlipid.pl; 5 Institute of Dietetics, Academy of Business and Health Science, 90-361 Łódź, Poland; a.kwasny@wsbinoz.pl; 6 Department of Medical Biochemistry, Wrocław Medical University, 50-368 Wrocław, Poland; lukasz.lewandowski@umw.edu.pl

**Keywords:** HFrEF, heart failure, nutritional status, obesity, BMI

## Abstract

Heart failure (HF) is a major health issue, affecting up to 2% of the adult population worldwide. Given the increasing prevalence of obesity and its association with various cardiovascular diseases, understanding its role in HFrEF outcomes is crucial. This study aimed to investigate the impact of obesity on in-hospital mortality and prolonged hospital stay in patients with heart failure with reduced ejection fraction (HFrEF). We conducted a retrospective analysis of 425 patients admitted to the cardiology unit at the University Clinical Hospital in Wroclaw, Poland, between August 2018 and August 2020. Statistical analyses were performed to evaluate the interactions between BMI, sex, and comorbidities on in-hospital mortality. Significant interactions were found between sex and BMI as well as between BMI and post-stroke status, affecting in-hospital mortality. Specifically, increased BMI was associated with decreased odds of in-hospital mortality in males (OR = 0.72, 95% CI: 0.55–0.94, *p* < 0.05) but higher odds in females (OR = 1.18, 95% CI: 0.98–1.42, *p* = 0.08). For patients without a history of stroke, increased BMI reduced mortality odds (HR = 0.78, 95% CI: 0.64–0.95, *p* < 0.01), whereas the effect was less pronounced in those with a history of stroke (HR = 0.89, 95% CI: 0.76–1.04, *p* = 0.12). In conclusion, the odds of in-hospital mortality decreased significantly with each 10% increase in BMI for males, whereas for females, a higher BMI was associated with increased odds of death. Additionally, BMI reduced in-hospital mortality odds more in patients without a history of cerebral stroke (CS) compared to those with a history of CS. These findings should be interpreted with caution due to the low number of observed outcomes and potential interactions with BMI and sex.

## 1. Introduction

Heart failure (HF) is a major health issue, affecting up to 2% of the adult population worldwide [1]. In 2017, there were approximately 64.3 million cases of HF globally. Among the different types of heart failure, heart failure with reduced ejection fraction (HFrEF) is particularly concerning as it leads to frequent hospitalizations and substantial mortality among cardiac patients [1,2,3,4,5]. Despite advances in pharmacological treatments and medical techniques, the prognosis for HFrEF patients remains uncertain, especially in the context of coexisting obesity [6,7,8].

Obesity, defined as a body mass index (BMI) of 30 kg/m^2^ or higher, is widely recognized as a significant risk factor for various cardiovascular diseases, including heart failure [9,10,11,12]. The mechanisms through which obesity affects the heart are complex, involving chronic inflammation, endothelial dysfunction, and metabolic alterations such as insulin resistance [13,14,15,16]. Furthermore, obesity is associated with a higher risk of developing other comorbid conditions, such as diabetes, hypertension, and chronic kidney disease, which further complicate the course of HFrEF [9,10,13].

The impact of obesity on the development of CVD, including heart failure (HF), remains undeniable. However, recent studies emphasize its more frequent association with HFpEF. It should be noted that, among patients with heart failure with reduced ejection fraction (HFrEF), obesity can influence various factors, including hemodynamics, remodeling, functional class according to the New York Heart Association (NYHA), the number of complications, and clinical outcomes [17]. The impact of obesity on the nature and severity of HFrEF symptoms can also affect the patient’s quality of life [17,18]. There are increasing reports indicating that interventions for morbid obesity coexisting with HFrEF can influence clinical parameters, including ejection fraction (EF). However, a crucial factor in determining the appropriateness of these interventions seems to be the definition of the purposefulness of weight loss in this patient group and distinguishing it from unintentional weight loss [11,17,19].

There is, however, a phenomenon known as the “obesity paradox”, suggesting that, in certain populations of patients with heart disease, a higher BMI may be associated with better clinical outcomes compared to those with lower BMI [12,20]. Nevertheless, one meta-analysis suggests that the obesity paradox should no longer be considered a significant prognostic factor [21,22].

Increasing attention has been given to sex differences in HF patients [23]. Sex can influence the risk of developing HF, the type of HF, and the progression of the disease [24]. Differences may exist in symptomatology, pathophysiology, diagnostics, functional status, as well as in the prescribed treatments and prognosis of patients, which warrants further investigation [25,26].

Given the increasing prevalence of obesity and the high incidence of HFrEF, it is essential to explore how obesity impacts clinical outcomes in heart failure patients, particularly in the context of sex differences and other risk factors [27,28,29]. Understanding these implications can lead to more effective management strategies for this growing patient population. Recent studies suggest that obesity may influence hospital stay duration and mortality rates in HFrEF patients, but the exact nature of this relationship remains unclear due to conflicting evidence and potential confounding factors.

This study aims to assess how body mass index (BMI) affects clinical outcomes, including length of hospital stay and in-hospital mortality, in patients with heart failure with reduced ejection fraction (HFrEF). The analysis will focus on identifying potential interactions between BMI and other risk factors, such as sex, age, and comorbidities, to better understand how these factors influence patient prognosis.

## 2. Materials and Methods

### 2.1. Study Design

This retrospective study involved analyzing medical records of patients who were admitted to the cardiology unit at the University Clinical Hospital in Wroclaw, Poland, due to heart failure with reduced ejection fraction (HFrEF). The data collection period spanned from August 2018 to August 2020. This study followed the STROBE (Strengthening the Reporting of Observational Studies in Epidemiology) guidelines to ensure the reliability and clarity of the observational study reporting.

### 2.2. Study Population and Data

We analyzed all patients who met the inclusion criteria: diagnosis of HFrEF and age ≥ 18 years. The final analysis included data from 425 patients. Key data points included patient demographics (age, sex), body mass index (BMI), length of hospital stay (LOHS), and laboratory results, such as total cholesterol (TC), high-density lipoprotein (HDL), low-density lipoprotein (LDL), triglycerides (TG), and albumin. Additional information gathered encompassed the type of heart failure (HF), New York Heart Association (NYHA) classification, and comorbidities (chronic kidney disease (CKD), arterial hypertension (HT), diabetes mellitus (DM), cerebral stroke (CS), and thyroid disease (TD)). Blood samples for laboratory tests were collected by a nurse at the time of admission. The nutritional status of the patients was assessed using the NRS-2002 questionnaire, with scores ≥ 3 indicating a risk of malnutrition. The World Health Organization (WHO) criteria were used to classify the patients into different BMI categories: underweight (BMI < 18.5 kg/m^2^), normal weight (BMI 18.5–24.9 kg/m^2^), pre-obese (BMI 25–29.9 kg/m^2^), and obese (BMI ≥ 30 kg/m^2^). Additionally, the patients were grouped into non-obese (BMI < 30 kg/m^2^) and obese (BMI ≥ 30 kg/m^2^) categories for auxiliary assessment of variable differences. Prolonged stay was defined as being hospitalized for a count of days higher than 10 (the 3rd quartile of hospitalization days in the population sample). All data were meticulously recorded in the patients’ medical documentation upon admission.

### 2.3. Statistical Analysis

Data pre-processing and statistical analysis were performed in Statistica 13.3 on the license of Wroclaw Medical University. Population sample characteristics in which both sexes were compared utilized two classic tests: the Mann–Whitney U test (continuous data) and the χ^2^ test (categorical data). The non-parametric approach in comparing the continuous data between groups was chosen based on the amount of missing data in some variables and the fact that not all parameters followed the normal distribution (as visualized with the Q-Q plots). Odds analysis was performed with the use of logistic regression. Its main purpose was to explore 2nd-order interactions between the variables (e.g., the impact of variable A on the way in which variable B modulates the analyzed odds, marked as ‘A × B’). Firstly, a set of variables was chosen a priori to the analysis. Secondly, all of the possible interactions were tested with the likelihood ratio (LR) type 1 test—comparing a model with each interaction to the naïve model in terms of informativeness. Selected interactions for which LR1 *p* < 0.05 were then analyzed in a full factorial model (e.g., containing terms ‘A’, ‘B’, and an interaction term ‘A*B’). Based on good practice standards in data modeling, discretization of the continuous variables was not performed, since the variables met the assumptions of the used method. Linearity vs. log(odds) was analyzed based on plots and the Box–Tidwell test). In cases when the raw data did not meet this linearity assumption, the variables were transformed and re-checked for meeting the assumption. An interaction that contained a continuous variable (BMI) was visualized with the Python 3.10.7 matplotlib package.

## 3. Results

### 3.1. Population Characteristics

Sex-related differences were observed in the contexts of age (*p* = 0.013), TG (*p* = 0.011), HDL-Chol (*p* = 0.031), TChol (*p* = 0.024), and incidence of CKD (*p* = 0.043). All of these factors were higher in female patients (Table 1).

The participants, likewise, showed differences in regard to their survival status (Table 2). Non-survivors were characterized by higher age (*p* < 0.001), BNP (*p* < 0.001), and hsCRP (*p* < 0.001) and a longer hospital stay (*p* < 0.001). Moreover, cholesterol concentrations (TChol, LDL-Chol, HDL-Chol) were lower among this group (*p* values: *p* = 0.004, *p* = 0.016, and *p* < 0.001, respectively). Non-survivors more frequently showed a higher NYHA class (*p* < 0.001). BMI values over 30 were spotted less frequently among them (*p* < 0.001), and they more frequently showed values of NRS ≥ 3 (*p* < 0.001).

### 3.2. Insights into the Two-Way Modulation of the Odds of Death—Based on Interaction Analysis

**Figure 1:**
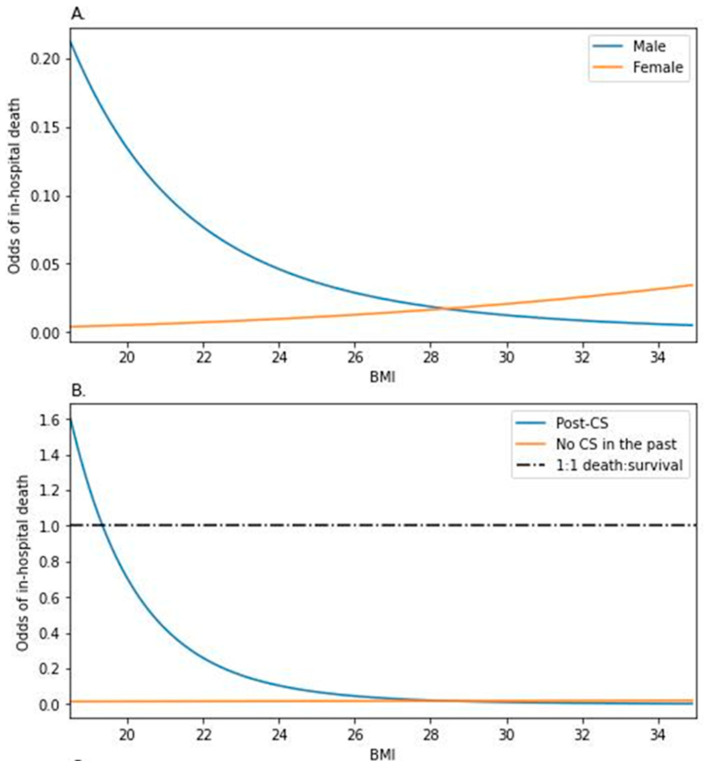
Modulation of the odds of death by (**A**) BMI and sex; (**B**) the post-CS status and BMI among the HFrEF participants.

Three interactions proved to be significant in the course of the data analysis—sex and BMI (LR1 *p* = 0.008, Figure 1A), post-CS and BMI (LR1 *p* = 0.012, Figure 1B). Upon being featured in the full factorial model, only the first two remained influential (Table 3), as indicated further in the text.

### 3.3. Interaction between Sex and BMI

The estimated odds of death of a male individual with BMI 28.4 (median value in the population sample) were 0.017 (*p* < 0.001, Table 3A). In relation to BMI 28.4, each 10% increase in BMI would decrease these odds by 74.83% (*p* = 0.003). Women of the same BMI did not significantly differ in the odds (*p* = 0.129), although an increase in BMI affected the odds among them differently—increasing the odds by 37.79% [OR = exp(−0.558 + 0.879), *p* = 0.010] with each 10% increase in BMI in relation to BMI 28.4. This phenomenon led to an approximately 2.408-fold increase in the female-to-male ratio of the odds for every 10% BMI increase in relation to BMI 28.4 (*p* = 0.010).

Thus, the answer to whether the mortality was higher in men compared to women was contextual (Figure 1A). While, among the patients with obesity, women would show higher odds of death compared to men, these odds would be markedly higher in men in the BMI 18.5–25.0 range.

### 3.4. Interaction between the Post-CS Status and BMI

The estimated odds of death of an individual with BMI 28.4 were 0.018 (*p* = 0.003, Table 3B), regardless of experiencing CS in the past (*p* = 0.685). Each 10% increase in BMI over 28.4 modulated the odds differently, depending on the post-CS status (2.581-fold increase in the non-CS/post-CS odds ratio, *p* = 0.046). The odds dropped by 72.93% (per 10% increase in BMI, *p* = 0.024) among the non-CS individuals, while the post-CS ones showed a 5.74% decrease [1/exp(−1.004 + 0.948), *p* = 0.046].

Based on the visualization of the statistical model (Figure 1B), one could argue about the contrast in the odds between the individuals of different CS statuses among the population with obesity. However, the odds ratio (post-CS/non-CS) rose as the BMI lowered, favoring the non-CS population for which the odds of death were constant in regard to BMI.

### 3.5. Insights into the Two-Way Modulation of the Odds of Prolonged In-Hospital Stay by BMI and the Post-CS Status

**Figure 2:**
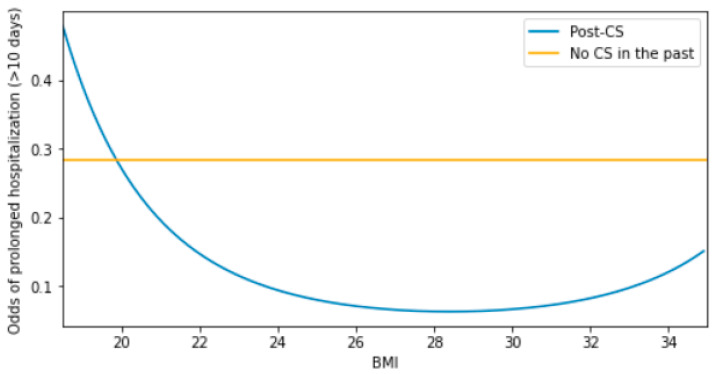
Pairwise modulation of the odds of a prolonged in-hospital stay (>10 days) by BMI and the post-CS status.

The interaction between BMI and the post-CS status was tested as significant by both the LR type 1 test (*p* = 0.018) and Wald test upon being featured in a full factorial model (*p* = 0.041, Table 4). The odds of a prolonged stay for a non-CS individual of 28.4 BMI (‘baseline’ individual) were estimated to be 0.284 (*p* < 0.001). A hypothetical non-CS individual of a different BMI would show the same odds (*p* = 0.166) compared to the baseline individual. However, an individual with a past CS experience and the same BMI as the baseline individual would show relatively lower odds (by 4.484-fold). This phenomenon stems from the finding that the odds in the post-CS stratum were significantly affected by BMI (*p* = 0.041). Based on the visualization of this classification model (Figure 2), one could assume that the impact of BMI on the odds, in the post-CS individuals, was positive among the patients with obesity or negative among individuals of BMI within its reference range (18.5–25.0).

## 4. Discussion

In our study, we observed significant interactions between sex and BMI that influenced in-hospital mortality among patients with HFrEF. Specifically, obesity was associated with better survival among men but not among women. Additionally, we found that the interaction between BMI and post-cerebral stroke (CS) status significantly influenced the odds of a prolonged in-hospital stay.

In our study, women represented less than 25% of the cohort (90 out of 425 patients). This low representation of females can be explained by several factors. One major factor is that men have a higher risk of developing HFrEF compared to women, primarily due to their higher likelihood of experiencing coronary artery disease (CAD) and myocardial infarction (MI), which are common precursors to HFrEF [30,31]. A large prospective observational study, which followed 28,820 patients for a median of 12 years, found that men had almost twice the risk of developing HFrEF compared to women [32]. Supporting these findings, data from the Swedish Registry “SwedeHF” showed that women constituted 55% of patients with HFpEF but only 29% of those with HFrEF [33]. Similar trends were observed in Polish research. In a study by Kałużna-Oleksy et al., only 19% of the study population were women [34].

Obesity is a significant risk factor for HF (OR = 1.69; 95% CI: 1.57–1.82) [27]. While obesity increases the risk of developing HF, it appears to exert a protective effect in patients already diagnosed with HF, a phenomenon known as the “obesity paradox”. This paradox has been observed in patients with HFrEF. The “obesity paradox” refers to the observation that patients with certain existing diseases, including HF, tend to have a better clinical course if they are overweight or have obesity [20,35]. Recently, the “obesity paradox” has been questioned, particularly in HFrEF patients like those in our study. In the study by Butt et al., 8399 HFrEF patients from the PARADIGM-HF trial were analyzed. Using crude BMI, the “obesity paradox” was observed for cardiovascular death and all-cause mortality. However, after adjusting for multiple factors, the paradox disappeared. When considering the waist-to-height ratio (WHtR), there was no evidence of the “obesity paradox” for hospitalization due to HF exacerbation, cardiovascular death, or all-cause mortality [21].

In other analyses of the PARADIGM-HF study, the “obesity paradox” was also not observed [36]. The apparent “obesity paradox” has been further challenged by more comprehensive data analyses in other studies. For example, a study by Fröhlich et al. involving 2936 patients with HFrEF assessed the relationship between BMI-classified body weight and mortality. While the “obesity paradox” was initially observed, it became much less pronounced after accounting for insulin treatment and completely disappeared in patients with poorly controlled glycemia (HbA1C percentage > 7.5%) [37]. Thus, in these studies involving patients with HFrEF, a thorough analysis of patient characteristics (with more comprehensive adjustments for other prognostic variables) and the use of alternative anthropometric indices refuted the “obesity paradox”. It appears, therefore, that the “obesity paradox” should be approached with caution. In light of the latest reports, the observed phenomenon can be more accurately described as the “BMI paradox”. Furthermore, it is worth noting that earlier studies on the “obesity paradox” did not include other prognostic variables, and investigating these may be crucial in understanding the mechanisms underlying this phenomenon [18]. It is sex, aging, nutritional status, and different therapeutic strategies that may contribute to the occurrence of the “obesity paradox”, highlighting the complex relationship between obesity and HF outcomes [38,39].

Several factors may significantly influence the observed “obesity paradox”: (1) BMI is an imprecise measurement, and the ratio of lean mass to fat mass is more important, as increased muscle mass protects against circulatory complications; (2) many studies did not account for physical activity, which significantly affects the risk associated with excess body weight; (3) age profile is important, as older people are less likely to have obesity, making younger age a potential protective factor rather than higher body weight; (4) reverse causality, such as increased risk in frail patients; (5) smoking and cancer can reduce body weight and blur causal relationships; (6) better nutrition and body reserves in individuals with excess weight; (7) better drug tolerance in people with higher body weight; (8) protective cytokine profiles, mitochondrial adaptation, and other biological mechanisms; (9) longer follow-up times can obscure the “obesity paradox”, as observed in Kaplan–Meier estimates showing increased incidences of CHF and rehospitalization rates among patients with obesity [38].

In our study, the “obesity paradox” was found in men with HFrEF (OR = 0.72, 95% CI: 0.55–0.94, *p* < 0.05), but we did not find it in women (OR = 1.18, 95% CI: 0.98–1.42, *p* = 0.08). In studies that showed, based on BMI, the occurrence of the obesity paradox in patients with HFrEF, no difference was found depending on sex [40,41]. In our study, there were factors among the women that may be important in influencing the results. In our study, the women were older (*p* = 0.013); had higher concentrations (*p* = 0.011), HDL-chol (*p* = 0.031), and TChol (*p* = 0.024); and had a higher incidence of CKD (*p* = 0.043). A similar relationship has been demonstrated in other studies assessing sex differences in HFrEF. It has been shown that women have a higher prevalence of comorbidities, such as HT, DM, anemia, TD, depression, and CKD [42,43,44]. Numerous studies have also indicated that women may not receive optimal pharmacotherapy and experience more frequent adverse effects resulting from pharmacotherapy [43,45]. Although this theory requires further research, these variables may affect prognoses among women. In a study by Marta Kałużna-Oleksy et al., BMI was significantly higher in males (29.4 ± 5.3 vs. 25.9 ± 4.7; *p* < 0.001). However, impaired nutritional status assessed by various methods, including BMI, was not significantly associated with a worse prognosis. Multivariable analysis indicated that NYHA class, lower estimated glomerular filtration rate, higher levels of B-type natriuretic peptide (BNP), higher *N*-terminal fragment of proBNP, and higher uric acid were independent predictors of all-cause mortality, regardless of sex and age [34].

It is worth emphasizing that the occurrence of the “obesity paradox” in patients with HF is significantly modified depending on the level of physical activity (in patients with HF who are not physically active, the “obesity paradox” is not observed) [46]. Because BMI does not distinguish between fat mass, fat-free mass, and lean mass, individuals with similar body mass indices may have vastly different body composition. BMI index does not reflect the distribution of visceral fat tissue and does not assess muscle mass, much less muscle strength [47]. The distribution of visceral fat tissue and muscle strength significantly influence the prognosis of patients with cardiovascular disease [35,48]. It is worth emphasizing that the risk of all-cause mortality in patients with HF and CKD is higher among those with HFrEF (HR = 2.43 versus HR = 1.59) [49], which may explain the observed results in our study. Age is also a factor modulating the occurrence of the “obesity paradox” [50]. It is important to note that current medications, such as SGLT2 inhibitors, GLP-1 receptor agonists, and sacubitril/valsartan, which are often prescribed to individuals with higher BMI, significantly improve outcomes. The impact of these medications should also be considered when interpreting the results related to obesity and mortality in HFrEF patients [51,52]. Also, an important factor shaping the occurrence of the “obesity paradox” is the etiology of HF. In patients with ischemic HF etiology, there is no protective effect of obesity on their prognosis [53].

In our study, the results suggested that a more comprehensive assessment of patients’ characteristics, including history of stroke, might indicate that, in people with obesity, HFrEF, and a history of stroke, the “obesity paradox” is less pronounced. This indicates a more holistic assessment of prognosis rather than only through the prism of the imperfect BMI. Specifically, we observed that the interaction between BMI and post-CS status could influence the length of a hospital stay. For patients without a history of CS, a higher BMI was associated with a shorter hospital stay. In contrast, for patients with a history of CS, a higher BMI did not significantly reduce the hospital stay, suggesting that post-CS status might modulate the impact of BMI on hospitalization outcomes. This observation aligns with previous studies showing that stroke survivors often have a higher burden of comorbidities and worse overall prognosis, which can influence the effect of BMI on hospital outcomes [54,55]. Other studies have highlighted that stroke patients typically experience higher rates of complications and prolonged recovery periods, which can diminish the benefits of a higher BMI observed in non-stroke populations [56,57]. Considering the low number of deaths observed in the female subpopulation (only six deaths) in our study, it is difficult to draw definitive conclusions about the impact of sex on mortality outcomes. The limited sample size and interaction with BMI suggest that the conclusions regarding the influence of sex and post-CS status should be interpreted with caution.

Survival rates at 1 and 5 years in HFrEF, HFmrEF, and HFpEF were 81%, 84%, 84%, and 47%, 61%, and 59%, respectively [58]. In the real-world setting, one in six patients with HFrEF develop worsening HF within 18 months of HF diagnosis. These patients have a high risk for 2-year mortality and recurrent HF hospitalizations [59]. Patients with HFrEF who had a worse prognosis were more likely to have hypertension, coronary artery disease, lipid disorders, atrial fibrillation, diabetes, acute coronary syndrome, chronic obstructive pulmonary disease, anemia, peripheral artery disease, depression, thromboembolism, stroke, and sleep apnea [59]. This shows that predicting prognosis based on BMI is completely pointless. The old slogan “people with obesity live shorter lives” in the eyes of a cardiologist in 2024 has not lost its relevance.

### 4.1. Practical Implication

However, it is crucial to approach the interpretation of BMI results with humility, particularly in patients with acute heart failure who may present with edema. Therefore, a holistic approach to patient assessment is necessary, one that goes beyond BMI and includes factors such as socioeconomic status, medication adherence, and detailed body composition analysis to provide a clearer and more comprehensive understanding of their health status.

Furthermore, the findings highlight the necessity for future research to focus on prospective studies that include comprehensive data collection on socioeconomic factors, medication adherence, and detailed body composition metrics. This will help validate and expand upon the findings of this study, ultimately leading to more effective and individualized treatment approaches for HFrEF patients.

By incorporating these practical implications into clinical practice, healthcare providers can enhance the management and outcomes of patients with HFrEF, tailoring interventions to better suit individual patient needs based on sex, comorbidities, and overall health status. Approaching the interpretation of BMI results with humility and considering the broader context of each patient’s condition will improve the precision and effectiveness of heart failure management strategies.

### 4.2. Study Limitation

The presented study had several limitations. One significant limitation was the relatively small subgroup of patients identified as being at risk of malnutrition, which comprised 4.3% of the study group (N = 425). Additionally, there were instances where NRS-2002 scores and BMI results were not recorded in the medical records, limiting the completeness of the data. Due to the retrospective nature of this study, we lacked access to data on socioeconomic status, medication adherence, and other potential confounding variables, which could have provided a clearer picture of the BMI-HFrEF relationship. Furthermore, this study did not include body composition analysis, and BMI, while commonly used, is not always a reliable indicator for assessing overweight and obesity. Measurements such as waist-to-hip ratio (WHR) or waist circumference, which provide better insights into central obesity, were not reported. Lastly, due to the anonymity of the medical records, it was not possible to assess the long-term survival of the heart failure patients. The medical records also did not include information regarding the patients’ prior treatments, such as the use of lipid-lowering drugs, which could have influenced the study outcomes. Moreover, the dataset used in this study was inadequate to explore interactions among the malnourished patients, since there were only 20 patients of NRS2002 ≥ 3. One could argue about the possible use of the NRS2002 variable as a continuous one—not categorical, although the characteristics of this scale (being a scoring one) would render the results hard to interpret (comparing NRS2002 = 1 patient with NRS2002 = 2 patients, etc.). Therefore, the idea of featuring the malnourished patients as an independent data subset for analysis was discarded.

These limitations suggest that further research, particularly prospective studies with comprehensive data collection and long-term follow-up, is necessary to validate and expand upon the findings of this study.

## 5. Conclusions

This study revealed significant interactions between sex and body mass index (BMI) affecting in-hospital mortality in patients with heart failure with reduced ejection fraction (HFrEF). Specifically, the odds of in-hospital mortality decreased significantly with each 10% increase in BMI for males, while for females, the increase in BMI was associated with higher odds of death. This finding highlights the complex interplay between sex and obesity in influencing patient outcomes.

Additionally, our initial analysis suggested an interaction between BMI and history of stroke (CS) status. For patients without a history of CS, the odds of death decreased substantially with increasing BMI. In contrast, for those with a post-CS status, the effect of increasing BMI on mortality was less pronounced.

While these results underscore the importance of considering both sex and BMI when assessing the prognosis of HFrEF patients, they should be interpreted with caution due to the low number of observed outcomes and potential interactions with BMI and sex. Further prospective studies with larger and more diverse populations are warranted to validate and expand upon the observed associations and to better inform clinical practice.

## Figures and Tables

**Table 1:** Differences in selected parameters between female and male participants of the HFrEF type of heart failure.

HFrEF (N = 425)									*p*
Variable (Quant.)	Female (n = 90)				Male (n = 335)				
	n	Median	25%	75%	n	Median	25%	75%	
Age	90	70.00	62.00	79.00	335	66.00	59.00	74.00	0.013
NT-pro-BNP [pg/mL]	19	5550.00	2166.40	9433.00	75	6976.00	2716.50	16,851.00	0.556
BNP [pg/mL]	68	712.00	209.50	1852.25	243	691.20	217.20	1421.40	0.529
TG [mg/dL]	87	124.00	91.00	168.00	318	103.00	84.00	138.00	0.011
LDL-Chol [mg/dL]	84	91.00	72.50	114.50	315	83.00	65.00	113.00	0.050
HDL-Chol [mg/dL]	85	44.00	33.00	51.00	316	38.00	31.00	47.50	0.031
TC [mg/dL]	87	158.00	138.00	194.00	316	148.50	122.50	182.50	0.024
hsCRP [mg/L]	86	3.89	1.27	15.43	326	5.01	2.01	14.74	0.261
Albumin [g/dL]	26	3.40	2.70	3.90	79	3.40	2.90	3.70	0.605
LOHS [days]	90	6.50	4.00	10.00	335	7.00	4.00	10.00	0.946
Variable:category (qual.)	Observed (n)		Frequency (%)		Observed (n)		Frequency (%)		*p*
Mortality: Death	6		6.67		15		4.48		0.395
NYHA: 1	8		9.64		40		12.23		0.897
NYHA: 2	28		33.73		112		34.25		
NYHA: 3	23		27.71		90		27.52		
NYHA: 4	24		28.92		85		25.99		
BMI < 18.5 [kg/m^2^]	2		2.22		7		2.09		0.971
BMI 18.5–24.9 [kg/m^2^]	26		28.89		90		26.87		
BMI 25–29.9 [kg/m^2^]	30		33.33		120		35.82		
BMI ≥ 30 [kg/m^2^]	32		35.56		118		35.22		
CKD: Yes	45		50.00		128		38.21		0.043
HT: Yes	59		65.56		219		65.37		0.974
DM: Yes	33		36.67		157		46.87		0.084
CS: Yes	8		8.89		41		12.24		0.377
MI: Yes	33		36.67		127		37.91		0.829
NRS2002 < 3	85		94.44		320		95.52		0.668
NRS2002: ≥3	5		5.56		15		4.48		

‘25%’ and ‘75%’ columns feature the 1st and 3rd quartiles, respectively. Abbreviations: n, number of participants; BMI, body mass index; LOHS, length of hospital stay; TC, total cholesterol; HDL, high-density lipoprotein; LDL, low-density lipoprotein; TG, triglycerides; hsCRP, high-sensitivity *C*-reactive protein; HF, heart failure; HFrEF, heart failure with reduced ejection fraction; NYHA, New York Heart Association classification; CKD, chronic kidney disease; HT, arterial hypertension; DM, diabetes mellitus; CS, cerebral stroke; NRS-2002, Nutritional Risk Score 2002.

**Table 2:** Differences in selected parameters between survivors and non-survivors of the HFrEF type of heart failure.

HFrEF (N = 425)									*p*
Variable (Quant.)	Survival (N = 404)				Death (N = 21)				
	n	Median	25%	75%	n	Median	25%	75%	
Age	404	66.00	59.00	74.00	21	77.00	72.00	84.00	<0.001
NT-pro-BNP [pg/mL]	94	6765.90	2716.50	16,611.00	0	-	-	-	-
BNP [pg/mL]	293	647.10	207.40	1334.20	18	1988.10	1251.50	3508.00	<0.001
TG [mg/dL]	388	106.00	86.00	144.50	17	117.00	82.00	157.00	0.739
LDL-Chol [mg/dL]	382	85.50	67.00	114.00	17	72.00	39.00	86.00	0.016
HDL-Chol [mg/dL]	384	39.50	32.00	48.50	17	25.00	21.00	36.00	<0.001
TC [mg/dL]	386	152.00	127.00	185.00	17	128.00	97.00	140.00	0.004
hsCRP [mg/L]	392	4.56	1.62	13.14	20	29.06	6.75	80.21	<0.001
Albumin [g/dL]	91	3.50	3.10	3.80	14	2.80	2.50	3.10	<0.001
LOHS [days]	404	6.00	3.00	10.00	21	13.00	6.00	16.00	<0.001
Variable:category (qual.)	Observed n		Frequency (%)		Observed n		Frequency (%)		*p*
Sex: Male	320		79.21		15		71.43		0.395
NYHA: 1	48		12.24		0		0.00		<0.001
NYHA: 2	138		35.20		2		11.11		
NYHA: 3	109		27.81		4		22.22		
NYHA: 4	97		24.74		12		66.67		
BMI < 18.5	6		1.49		3		14.29		<0.001
BMI 18.5–24.9	111		27.48		5		23.81		
BMI 25–29.9	141		34.90		9		42.86		
BMI ≥ 30	146		36.14		4		19.05		
CKD: Yes	166		41.09		7		33.33		0.481
HT: Yes	264		65.35		14		66.67		0.901
DM: Yes	180		44.55		10		47.62		0.783
CS: Yes	45		11.14		4		19.05		0.269
MI: Yes	150		37.13		10		47.62		0.333
NRS2002	389		96.29		16		76.19		<0.001
NRS2002: ≥3	15		3.71		5		23.81		

‘25%’ and ‘75%’ columns feature the 1st and 3rd quartiles, respectively. Abbreviations: n, number of participants; BMI, body mass index; LOHS, length of hospital stay; TC, total cholesterol; HDL, high-density lipoprotein; LDL, low-density lipoprotein; TG, triglycerides; hsCRP, high-sensitivity *C*-reactive protein; HF, heart failure; HFrEF, heart failure with reduced ejection fraction; NYHA, New York Heart Association classification; CKD, chronic kidney disease; HT, arterial hypertension; DM, diabetes mellitus; CS, cerebral stroke; NRS-2002, Nutritional Risk Score 2002.

**Table 3:** Relation between sex, BMI, and post-CS status and the odds of in-hospital death—based on the exploration of meaningful (LR1 *p* < 0.05) interactions.

A. Full factorial model with sex and BMI (LR1 *p* = 0.008)							
Variable	Category	β_i_	β_i_ SE	β_i_ −95% CI	β_i_ 95% CI	Estimate	*p*
Intercept (baseline value)	-	−4.061	0.502	−5.046	−3.077	0.017	<0.001
Sex	Female	1.148	0.756	−0.333	2.629	-	0.129
log1.1(BMI) (centered at BMI 28.4)	-	−0.558	0.188	−0.927	−0.189	0.572	0.003
Sex*log1.1(BMI)	-	0.879	0.342	0.209	1.548	2.408	0.010
B. Full factorial model with post-CS and BMI (LR1 *p* = 0.012)							
Variable	Category	β_i_	β_i_ SE	β_i_ −95% CI	β_i_ 95% CI	Estimate	*p*
Intercept (baseline value)	-	−4.042	1.359	−6.706	−1.378	0.018	0.003
post-CS	Non-CS	0.567	1.398	−2.173	3.307	-	0.685
log1.1(BMI) (centered at BMI 28.4)	-	−1.004	0.443	−1.873	−0.135	0.366	0.024
post-CS*log1.1(BMI)	-	0.948	0.475	0.018	1.879	2.581	0.046

Abbreviations: LR1, likelihood ratio type 1 test; SE, standard error; CI, confidence interval; BMI, body mass index; CS, cerebral stroke.

**Table 4:** Relation between post-CS status and BMI and the odds of in-hospital death—based on the exploration of meaningful (LR1 *p* < 0.05) interactions.

Variable	Category	β_i_	β_i_ SE	β_i_ −95% CI	β_i_ 95% CI	Estimate	*p*
Intercept (baseline value)	-	−1.258	0.143	−1.539	−0.978	0.284	<0.001
post-CS	Post-CS	−1.502	0.750	−2.973	−0.032	0.223	0.045
BMI^2^	-	0.002	0.002	−0.001	0.006	-	0.166
post-CS*BMI^2^	-	0.021	0.010	0.001	0.040	1.021	0.041

Centering refers to variable transformation in which the median value from the population is subtracted from all the raw values of a variable. The ‘Estimate’ column refers to odds (in the case of analyzing the intercept), odds ratio (in the case of analyzing the effects), or the ratio of odds ratios (in the case of analyzing the interactions). Abbreviations: CI, confidence interval; LR1, likelihood ratio type 1 test; SE, standard error; BMI, body mass index; CS, cerebral stroke

## Data Availability

The data can be obtained by contacting the corresponding author.
